# Ovalbumin sensitization of guinea pig at birth prevents the ontogenetic decrease in airway smooth muscle responsiveness

**DOI:** 10.14814/phy2.12241

**Published:** 2014-12-11

**Authors:** Pasquale Chitano, Lu Wang, Simone Degan, Charles L. Worthington, Valeria Pozzato, Syed H. Hussaini, Wesley C. Turner, Delbert R. Dorscheid, Thomas M. Murphy

**Affiliations:** 1 Division of Pediatric Pulmonary and Sleep Medicine, Department of Pediatrics, Duke University Medical Center, Durham, North Carolina; 2 James Hogg Research Centre, Institute for Heart and Lung Innovation and Department of Medicine, University of British Columbia, Vancouver, British Columbia, Canada; 3 Duke Center for Molecular and Biomolecular Imaging, Duke University Medical Center, Durham, North Carolina; 4 Duke Department of Radiology, Duke University Medical Center, Durham, North Carolina

**Keywords:** Airway hyperresponsiveness, asthma, maturation, neonatal allergic sensitization, ontogenesis

## Abstract

Airway smooth muscle (ASM) displays a hyperresponsive phenotype at young age and becomes less responsive in adulthood. We hypothesized that allergic sensitization, which causes ASM hyperresponsiveness and typically occurs early in life, prevents the ontogenetic loss of the ASM hyperresponsive phenotype. We therefore studied whether neonatal allergic sensitization, not followed by later allergen challenges, alters the ontogenesis of ASM properties. We neonatally sensitized guinea pigs to ovalbumin and studied them at 1 week, 3 weeks, and 3 months (adult). A Schultz‐Dale response in isolated tracheal rings confirmed sensitization. The occurrence of inflammation was evaluated in the blood and in the submucosa of large airways. We assessed ASM function in tracheal strips as ability to produce force and shortening. ASM content of vimentin was also studied. A Schultz‐Dale response was observed in all 3‐week or older sensitized animals. A mild inflammatory process was characterized by eosinophilia in the blood and in the airway submucosa. Early life sensitization had no effect on ASM force generation, but prevented the ontogenetic decline of shortening velocity and the increase in resistance to shortening. Vimentin increased with age in control but not in sensitized animals. Allergic sensitization at birth without subsequent allergen exposures is sufficient to prevent normal ASM ontogenesis, inducing persistence to adulthood of an ASM hyperresponsive phenotype.

## Abstract (precis)

Airway smooth muscle (ASM) displays a hyperresponsive phenotype at young age and becomes less responsive in adulthood. In this study, we found that allergic sensitization at birth without subsequent allergen exposures is sufficient to prevent normal ASM ontogenesis, inducing persistence to adulthood of an ASM hyperresponsive phenotype.

## Introduction

There is increasing evidence that the first years of life are critical for the development of chronic asthma, as shown by elevated incidence of asthma during infancy/early childhood and by the observation at this age of functional alterations associated with the onset of asthma later in life (Kelly et al. 1990; Jenkins et al. 1994; Martinez 2001, 2009; Stern et al. 2008). For instance, reduced airway function and airway hyperresponsiveness in early childhood are strong predictors of persistent wheezing or later onset of asthma (Sears et al. 2003; Turner et al. 2004; Stern et al. 2008). Although relevant risk factors and early environmental insults associated with persistent asthma are mostly known (Martinez 2011), it is not entirely understood what causes this susceptibility in young individuals. It is possible that immature features of the airway physiology favor the onset of the disease in young subjects and the persistence of airway hyperresponsiveness to adulthood. Interestingly, healthy juveniles show some degree of greater airway responsiveness than adults both in human and animal species (Hopp et al. 1985; Becker et al. 1989; Montgomery and Tepper 1990; Tepper et al. 1995a, 1995b; Shen et al. 1996; Weist et al. 2002). We have shown that airway smooth muscle (ASM) function undergoes dramatic changes during normal ontogenesis. Thus, ASM displays a temporary multifactorial hyperresponsive phenotype at a young age that is characterized by increased shortening velocity, reduced relaxation, reduced passive stiffness, and increased force after mechanical oscillation (Chitano et al. 2000, 2002, 2005, 2007; Chitano and Murphy 2003; Wang et al. 2005a, 2005b, 2008). Our findings prompted us to suggest that the ASM hyperresponsiveness at young age is the manifestation of cellular characteristics that may confer a specific vulnerability to environmental insults, such as allergen exposure, which in turn result in persistence of the ASM hyperresponsive phenotype to adulthood (Chitano et al. 2007).

A number of studies have shown that ASM shortening capacity and velocity are increased in hyperresponsive airways (Antonissen et al. 1979; Rao et al. 1991; Jiang et al. 1994; Mitchell et al. 1994; Ma et al. 2002; Stephens et al. 2003), suggesting that altered ASM function may be a prominent component in the pathogenesis of asthma. In several animal models, airway hyperresponsiveness associated with airway inflammation and enhanced ASM contractility is attained by allergen sensitization and subsequent airway challenges with the sensitizing agent (Mitchell et al. 1993; Van Oosterhout et al. 1993; Lewis et al. 1994; ten Berge et al. 1995; de Boer et al. 2001; Moir et al. 2003; Maarsingh et al. 2006). When animals are studied without antigen challenge to model early stages of airway disease, the airway inflammation is not observed, but the ASM hyperresponsiveness is fully attained (Antonissen et al. 1979; Jiang et al. 1994; Stephens et al. 2003). This suggests that the ASM hyperresponsiveness is not secondary to airway inflammation, but a primary alteration in allergen‐sensitized animals.

We hypothesized that early allergen sensitization alters the normal ASM ontogenesis, thus inducing a persistent ASM hyperresponsiveness. In this study, we sought to investigate the effect of allergen sensitization during the first week of life on the ontogenesis of ASM mechanical properties. In order to exclusively study the role of the initial sensitization and limit the involvement of chronic inflammatory response, no allergen booster or challenge was used in this work. We also evaluated whether an inflammatory process was present in our model of early sensitization and whether the protein vimentin, which play a role in passive mechanical response, was affected. Our results show that allergic sensitization at birth induces the persistence of the immature ASM hyperresponsive phenotype to adulthood.

## Methods

### Animals and sensitization to ovalbumin

Hartley guinea pigs (Charles River Laboratories, Inc., Wilmington, MA) were employed for this investigation according to a protocol (Protocol Registry # A161‐08‐06) approved by the Duke University Institutional Animal Care and Use Committee. Animals were housed following the Institutional Policy of environmental enrichment, which regulates cage environment in order to enhance animal well‐being.

This study was conducted in the same three age groups used in our laboratory to study the ontogenesis of ASM function in healthy animals (Chitano et al. 2000, 2002, 2005). We used 1‐week‐old, as infant guinea pigs (1 week, 9.2 ± 0.7 [mean ± SD] day old, 160 ± 26 [mean ± SD] g, *n* = 32), 3‐week‐old, as juvenile guinea pigs (3 week, 24.9 ± 4.2 day old, 312 ± 46 g, *n* = 18), and 2‐ to 3‐month‐old, as adult guinea pigs (adult, 79.5 ± 18.9 day old, 571 ± 103 g, *n* = 23). Control animals used in this study were five infants (7.8 ± 1.0 day old, 186 ± 38 g), five juveniles (21.4 ± 1.1 day old, 218 ± 26 g), and five adults (80.8 ± 5.7 day old, 699 ± 46 g). Animals were anesthetized with 200 mg/kg Na‐pentobarbital i.p. to collect peripheral blood, tracheas, and lungs. Upon complete achievement of anesthesia, which was confirmed by absence of reflex in response to toe clamping, trachea and lungs were exposed, excised, and immediately immersed in Krebs‐Henseleit (K‐H) buffer solution aerated with 95% O_2_ and 5% CO_2_. The composition of the K‐H was the following (mmol/L): 115 NaCl, 25 NaHCO_3_, 1.38 NaH_2_PO_4_, 2.5 KCl, 2.46 MgSO_4_, 1.9 CaCl_2_, and 11.2 dextrose.

Ovalbumin was used as sensitizing agent according to the following protocol, which was devised to limit antigen injections to the first days of life to minimize chronic inflammation while providing an effective immune response characterized by systemic ovalbumin sensitization. A solution of 5 mg/mL of ovalbumin was prepared fresh in 1/1 incomplete Freund's adjuvant/0.9% NaCl. Guinea pigs were immunized with three subcutaneous injections of the ovalbumin solution (3 mg/kg b.w.) on three different sites on the animal back performed at birth and at intervals of 2 days. This sensitization protocol was not followed by any further exposure/challenge to ovalbumin, since we aimed at detecting potential changes in the ontogenetic progression of ASM function that could be exclusively attributed to the initial ovalbumin sensitization. In seven guinea pigs, we performed our sensitization protocol in adulthood in order to evaluate possible differences in comparison with neonatal sensitization. These animals were studied 2 weeks after the third ovalbumin injections. At the time of euthanasia, to test the occurrence of sensitization, a tracheal ring from each animal was mounted in an organ bath and challenged with 0.1 mg/mL ovalbumin, which produces a clear Schultz‐Dale response, that is, a substantial contraction, if the animal is sensitized (Sigurdsson et al. 1995; Chitano et al. 1996). In one subset of the 1‐week group, in which we found a significant number of rings that did not display a Schultz‐Dale response, the challenge was done with 1 mg/mL ovalbumin. Each ring was first contracted with 10^−3^ mol/L acetylcholine, which induces a submaximal contraction in ASM, and tension continuously recorded. When a steady‐state contraction was attained, rings were rinsed until tension returned to baseline resting values, and next exposed to ovalbumin. The tension at steady state generated by 10^−3^ mol/L acetylcholine was used as reference value to normalize the contractile response to ovalbumin.

We first performed the experiments on airway smooth muscle active and passive mechanical response as described below. Control animals were acquired, housed, and studied in parallel with the sensitized animals and this part of our investigation was completed between January 1997 and September 2000. While the data from sensitized animals were kept to be integrated with additional sets of experiments on inflammatory and structural component of our sensitization model, the data from those control experiments were published in 2000 and are therefore reported in this study as citations of our previously published article (Chitano et al. 2000). We decided not to perform additional control mechanical experiments to include in this manuscript considering the potential source of discrepancies represented by the significant time gap between new controls and old sensitized data and by the associated changes in experimenters, materials, and animal features. We also decided not to duplicate our mechanical experiments in control and sensitized animals, since it would have required substantial additional number of animals, costs, and time. We felt that the scientific benefits would have been minimal in comparison with the magnitude of the effort and costs. We thought that ethical considerations about the number of animals to be used had to be taken into account as well.

A set of control and sensitized guinea pigs of each age group was later used to collect peripheral blood and lungs to study IgE serum levels, as well as the occurrence of an inflammatory process, the ASM content in the airway wall, and the tissue content of vimentin (see next sections). Serum was prepared from part of the collected blood and levels of total IgE were measured by ELISA in control and sensitized animals as an additional indication of the efficacy of our sensitization protocol.

### Evaluation of the airway inflammation

In this sensitization model, being the animals unchallenged, ASM alterations are expected to occur in the absence of a chronic inflammatory process. This was evaluated in control and sensitized animals by analyzing the number of leukocytes in peripheral blood samples and the cellular infiltrate in lung tissue. Blood was immediately collected from the jugular vein and put in Vacutainer Blood Collection Tubes coated with EDTA‐K2 and diluted with Kimura staining solution at 1:10. The number of total leukocytes, eosinophils, neutrophils, and monocytes was measured with a hemocytometer at ×400 magnification and were expressed as number ×10^5^/mL blood. To study airway infiltration of inflammatory cells, one lung from each animal was distended via the main bronchus and fixed with formalin overnight at 20 cm H_2_O and then embedded in paraffin. Five‐micrometer‐thick cross sections were stained with hematoxylin–eosin (H&E) to quantify cellular infiltration. Cells were counted in the submucosa layer of the main airways at ×1000 magnification from five serial slides in each animal and expressed as number/mm^2^.

We evaluated the abundance and persistence of CD4+ lymphocytes in the airways by immunohistochemistry of lung sections (5 *μ*m) from lobes that were fixed in 4% formaldehyde and embedded in paraffin. Section were immunolabeled with a mouse monoclonal antiguinea pig CD4 antibody (1:10 dilution, clone CT7, AbD Serotec, Raleigh, NC). Antigen unmasking for CD4 was performed with trypsin. Nonspecific binding was blocked with 10% (v/v) normal horse serum followed by overnight primary antibody incubation at 4°C. Secondary antibody staining was performed for 30 min at RT with 1:200 biotinylated horse anti‐mouse IgG1 (Vectorlab, Burlingame, CA). Detection was performed with Vectastain ABC complex (Vectorlab) followed by DAB peroxidase reaction (DAB substrate kit for peroxidase, Vectorlab). Slides were finally counterstained with methyl green. For histological assessment, 10 images of large airways were randomly taken at ×400. The tissue around the airways was erased using Photoshop (Adobe) to assess the staining only in the airway wall. A quantitative analysis was carried out using a color thresholding method in ImageJ (version 1.45s, NIH, Bethesda, MD). Two macros were created to automatically calculate the area positive to the antibody and the total area of the airway tissue. To avoid biased assessment, the same macro was run for all the analyzed images. From each original photomicrograph two downstream thresholded images were automatically generated representing the stained and total area of the tissue. These images were used to visually verify the appropriate correlation of the stained and total area of the tissue with the original image. The antibody staining was calculated as the ratio between the stained area and total area of the airway tissue and expressed as percentage of the airway wall.

### Airway smooth muscle active mechanical properties

After cleaning away loose connective tissue, tracheal strips were obtained for the study of ASM mechanical properties. The preparation was performed in K‐H solution buffered to pH 7.35–7.45 with 95% O_2_/5% CO_2_. As previously described (Chitano et al. 2000, 2002), ~1‐mm‐wide strips with ~2 mm cartilaginous attachments at both ends were dissected from transverse sections of the trachea, such that the ASM was parallel oriented along the longitudinal axis of the strips. Care was taken to preserve the integrity of the epithelium. One cartilaginous end was clamped in a stainless steel clip at the bottom of an 80 mL double jacketed organ bath in 37°C K‐H. The other end was fixed to the transducer tip of an electromagnetic lever system with 4–0 braided silk surgical thread inserted through the cartilage. In such setting, the two cartilage pieces formed the holders of the muscle via their natural structural connections. We chose to use tracheal strips instead of bronchial strips even though heterogeneous ASM response along the airways has been shown and small central airways may be more relevant to airway hyperresponsiveness than large proximal airways. We showed that the force generation and the release of histamine in response to the sensitizing allergen were higher in small than large bronchi from ragweed‐sensitized dogs (Chitano et al. 1996). However, the differences were more quantitative than qualitative, thus not precluding the use of tracheal smooth muscle in mechanical studies. The force‐velocity features of airway smooth muscle from sensitized animals also differ from controls in a qualitatively similar fashion in tracheal and bronchial smooth muscle (Jiang et al. 1994). Although we are not aware of any study addressing the heterogeneity of airway smooth muscle contractility along the airways during ontogenesis, we can reasonably assume that the effects of neonatal sensitization on the mechanics of tracheal smooth muscle is indeed representative of the effects on the airways in general. Additionally, we needed to take into account practical considerations about the airway size, ASM orientation, and potential manipulation damage that could have affected the feasibility of force‐velocity experiments in bronchi from 1‐week‐old guinea pigs.

Tracheal strips were equilibrated in K‐H solution containing 10^−5^ mol/L indomethacin for 60 min. Indomethacin was used to abolish intrinsic tone, which in guinea pigs is extremely variable and would have affected the values of force measurements rendering ambiguous the interpretation of force generation data. Then, supramaximal electric field stimulation (EFS) (18 V, 60 Hz, 400 mA/cm^2^) was performed using platinum electrodes. The study was performed with a computerized electromagnetic lever system modified from its original design (Brutsaert et al. 1971) (Qjin Design, Winnipeg, MB, Canada) as previously described (Chitano et al. 2000, 2002). Strips were stretched and adapted to a length at which the maximal force was produced in response to EFS. Values of stress were calculated by normalizing force per cross‐sectional area (CSA) of the strip. CSA was obtained by measuring width and thickness of each strip through a VK‐C370 digital signal processor Hitachi video camera (Hitachi Home Electronics, Inc., Norcross, GA) and by dividing the calculated area by 1.35, a correction factor accounting for the geometry of the strip that we previously derived by morphometric analysis of guinea pig tracheal strips (Chitano et al. 2000). The maximum rate of stress generation was obtained as the maximum value of the derivative of the force recording.

Force–velocity (FV) curves were elicited using the quick‐release load‐clamp technique (Chitano et al. 2000). At 2.5 sec after the onset of the stimulus, when rapidly cycling cross‐bridges are active (Dillon et al. 1981), load‐clamps to various afterloads were applied by abruptly (within 3 msec) changing conditions from isometric to isotonic. After a rapid transient (~80 msec) due to shortening of the smooth muscle series elastic component, a slow transient was recorded and its maximal slope (~170 msec after the quick release) was computed and identified as the maximum velocity of shortening for each given afterload. Afterloads were applied randomly to avoid time‐dependent and history‐related effects on shortening velocity. The maximal velocity of shortening at zero load, *V*_0_, was calculated by fitting the FV curves obtained for each strip with a form of the Hill equation modified for ASM (Wang et al. 1994). As an index of contractility for conditions intermediate to zero load and isometric that may conceivably be regarded as physiological, we evaluated the maximum power developed by our tracheal strips. Power output, which is defined as work over time or force time velocity, is higher when the force–velocity curvature is reduced and reflects an increased fraction of attached force‐generating cross‐bridges (Seow 2013). Intuitively, increased power can be described as the ability of ASM to produce a severe bronchospasm in shorter time. We calculated power as the maximum value of the product of each applied afterload and the maximum velocity of shortening reached with that given afterload.

The content of ASM in control and sensitized airways at different stages of maturation was studied by immunohistochemistry in order to evaluate whether differences in ASM mechanical properties originated from potential alterations in ASM mass induced by allergen sensitization. ASM was visualized by IHC as *α*‐smooth muscle actin positive staining in lung sections from the same lobes described above for the study on CD4+ staining and measured as area of ASM over total airway wall area. Antigen unmasking was done with Triton‐X (Sigma, St Louis, MO). Following blocking with 10% normal horse serum, slides were incubated overnight at 4°C with a mouse monoclonal anti‐*α*‐smooth muscle actin alkaline phosphatase conjugate antibody 1:125 dilution (clone 1A4, Sigma). Detection was performed by alkaline phosphatase (Vectorlab) and slides counterstained with methyl green. For histological assessment, the same methods and criteria employed for CD4+ staining described above were used to quantify ASM in the airway wall.

### Airway smooth muscle passive mechanical properties

To evaluate passive mechanical properties of tracheal strips in our age groups, from the modified Hill's equation fittings described before for the calculation of shortening velocity, we calculated the value *α*·*γ*/*β*, an index of the internal resistance to shortening (RSi), where *α* and *β*/*γ* approximate to Hill's *a* and *b* constants, respectively (Seow and Stephens 1986; Wang et al. 1994). The constant *α* (mN/mm^2^) has units of force that in slowly contracting muscle (i.e., smooth muscle) is independent of the load, while *β*/*γ* (l_ref_/s) is a constant with units of velocity. This index of the RSi reflects all internal factors that increasingly reduce shortening velocity of unloaded muscle during contraction, that is, those intracellular components, such as cytoskeletal proteins, that are compressed during shortening.

In lung sections from the same lobes described above for CD4+ staining, we then evaluated by IHC the presence in the airways of the cytoskeletal protein vimentin, which has been shown to play an important role in tissue mechanical stability (Goldman et al. 1996; Eckes et al. 1998; Wang and Stamenovic 2000). After antigen retrieval with citrate buffer, pH 6.0, and blocking of nonspecific binding with 10% normal horse serum, slides were incubated overnight at 4°C with monoclonal mouse antivimentin antibody, which was ready to use (clone V9, AbD Serotec). As secondary antibody we used biotinylated horse anti‐mouse, 1:200 dilution for 30 min at room temperature. Detection was performed with ABC complex, followed by DAB peroxidase reaction and slides were counterstained with methyl green.

### Drugs and chemicals

Indomethacin were purchased from Sigma Chemical Co.; pentobarbital sodium from Abbot Laboratories, Chicago, IL; 10% neutral buffered formalin from Trend Scientific Inc., St. Paul, MN.

### Data analysis

Data are expressed as mean ± SEM, except when differently indicated. Data were compared using either Student's *t*‐test or analysis of variance (ANOVA). The least significant difference (LSD) post hoc analysis was used to find which groups were responsible for differences revealed by ANOVA. Statistical analysis was carried out using SigmaStat (V. 3.5, Systat, San Jose, CA). Values of *P* < 0.05 were considered as statistically significant.

## Results

### Sensitization to ovalbumin

**Figure 1.**
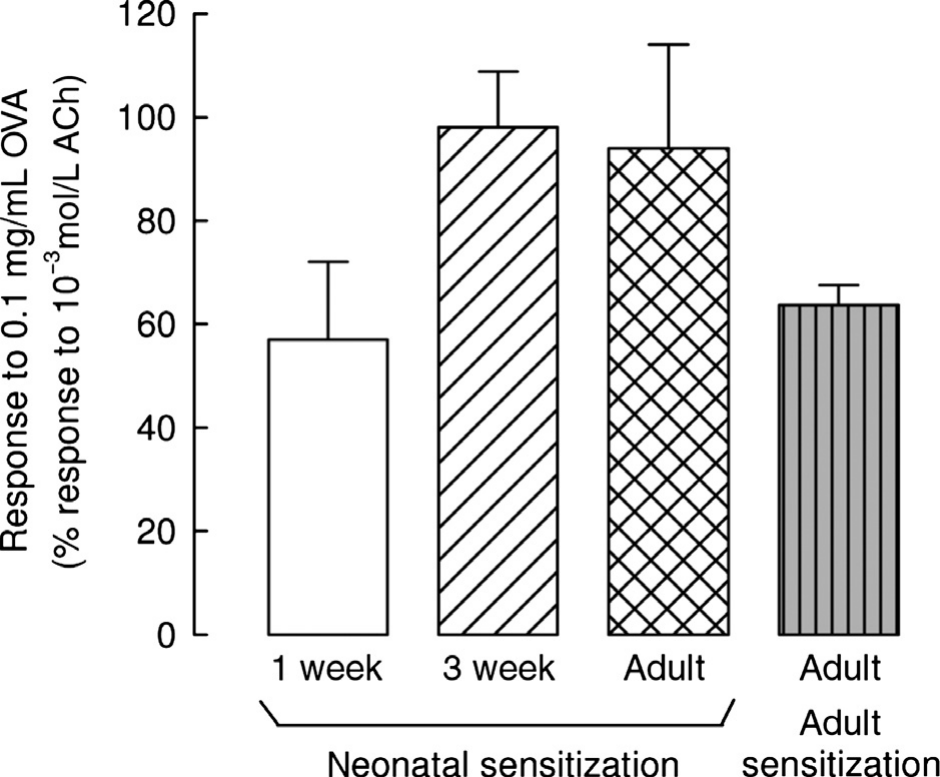
Contractile response to ovalbumin. Challenge of airway smooth muscle with the sensitizing agent induced a contractile response that shows the occurrence of allergic sensitization. In tracheal rings from different age guinea pigs sensitized at birth and from animals sensitized as adults, a substantial contraction was induced by the sensitizing agent ovalbumin (OVA). Means and standard errors are shown, *n* = 6, *n* = 16, and *n* = 9 for 1‐week, 3‐week, and adult animals neonatally sensitized, respectively; *n* = 7 for animals sensitized as adults.

The occurrence of sensitization to ovalbumin was tested in each animal by studying the contractile response, or Schultz‐Dale response, elicited in tracheal rings by 0.1 mg/mL ovalbumin (Fig. 1). Ovalbumin elicited no contractile response in rings from control animals, confirming the absence of a specific response in the tracheal tissue of animals that were not injected with this antigen. Conversely, ovalbumin induced a substantial contraction in the rings of all animals from the 3‐week and adult groups injected neonatally with ovalbumin, thus showing that our protocol results in sensitization of 100% of the treated animals. The extent of the response was comparable to the contractile response induced by 10^−3^ mol/L acetylcholine, which is equivalent to the maximal response that a given muscle can possibly produce, and did not decrease with age, suggesting that a permanent level of allergen sensitization is achieved in our model.

In the 1‐week group, we found that rings from about 50% of the animals, 8 of 14, did not respond to 0.1 mg/mL ovalbumin. Rings that displayed a contractile response to ovalbumin produced about 60% of the tension generated by rings from the 3‐week and adult groups, although the difference was not statistically significant. To test whether this lower response was the maximal contraction that could be produced by ovalbumin at this age/stage of sensitization, in an additional group of 1‐week‐old ovalbumin‐injected animals, we exposed tracheal rings to a tenfold concentration of ovalbumin, that is, 1 mg/mL. Also, in this group, we observed a Schultz‐Dale response in about 50% of the animals: rings from nine guinea pigs were nonresponders, whereas in rings from seven animals ovalbumin induced a response of 41.1 ± 9.8% of the one produced by 10^−3^ mol/L acetylcholine.

ASM contraction determined by ovalbumin in tracheal rings from animals that underwent the sensitization protocol at the adult age was lower, although not significantly different, compared with the 3‐week and the adult animals sensitized neonatally. The Schultz‐Dale response was observed in all rings from animals injected as adults.

**Figure 2.**
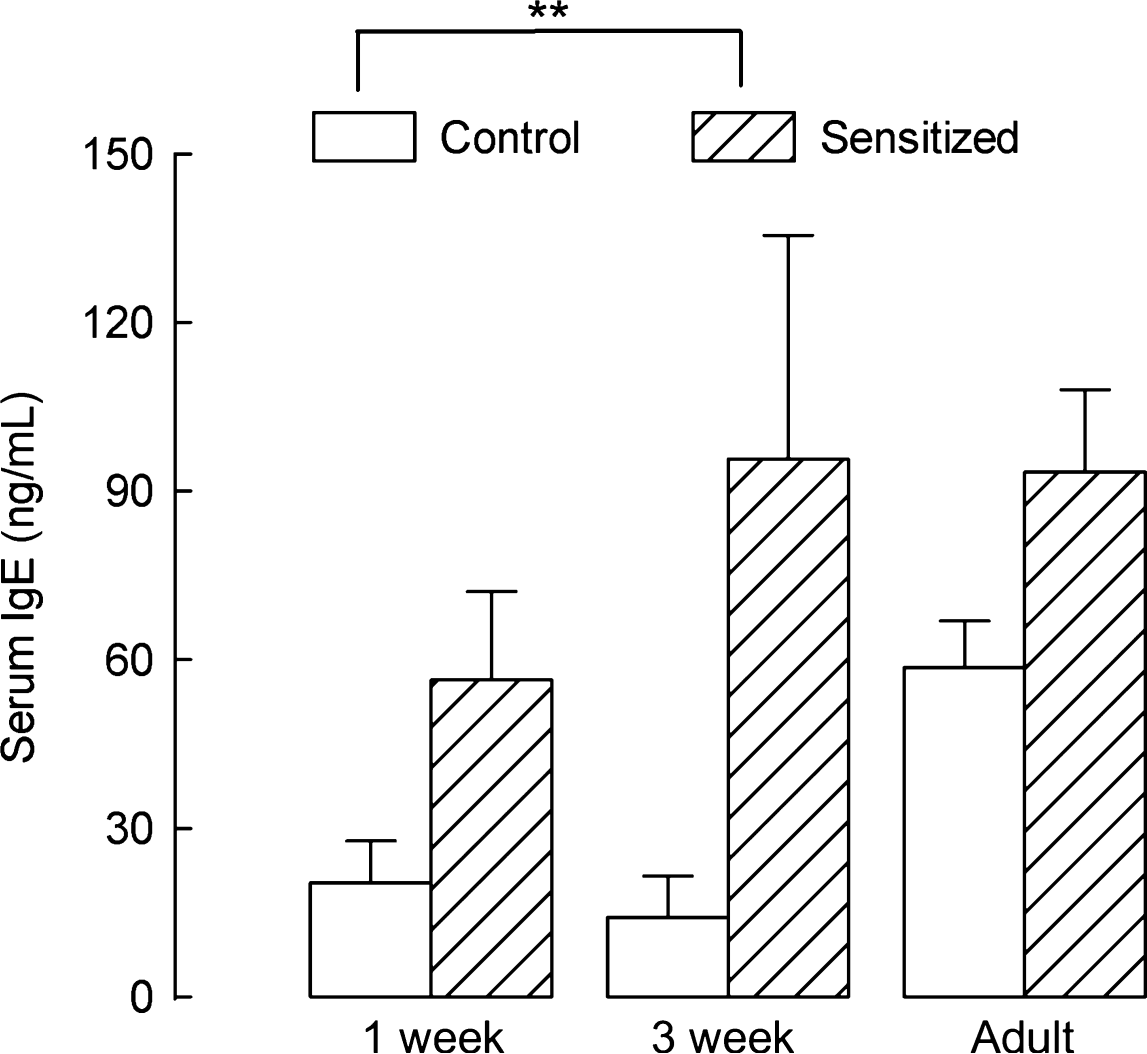
Serum IgE titers. Elevation of serum IgE titers confirmed the efficacy of our sensitization protocol. Total IgE titers were measured by ELISA in serum from different age control guinea pigs and from animals sensitized at birth. The effect of sensitization was statistically significant, *P* < 0.01 by ANOVA. Means and standard errors are shown, *n* = 4, *n* = 5, and *n* = 5 for 1‐week, 3‐week, and adult control animals, *n* = 5, *n* = 6, and *n* = 5 for 1‐week, 3‐week, and adult‐sensitized animals, respectively.

To further evaluate the efficacy of our sensitization protocol, we measured the values of serum IgE levels, which were significantly increased in sensitized compared with control animals (Fig. 2, *P* < 0.01 by ANOVA).

### Occurrence of inflammation

**Figure 3.**
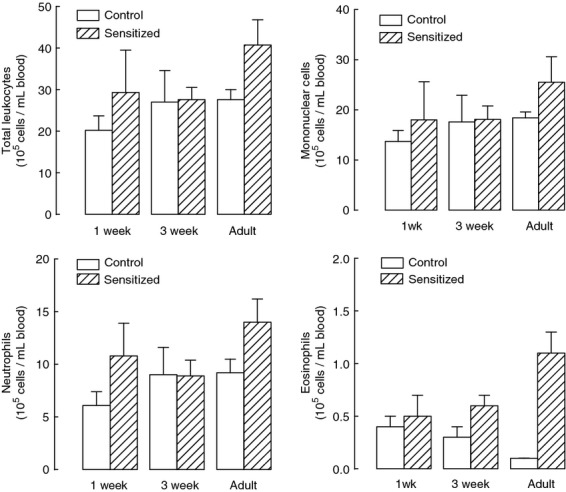
Blood cell counts. The number of leukocytes in the blood was not affected by ovalbumin sensitization, except for an increased number of eosinophils in adults neonatally sensitized compared with control animals. Blood leukocytes were measured by Kimura staining in different age control guinea pigs and in animals sensitized at birth. Blood eosinophils were significantly increased in sensitized adults compared to controls (*P* < 0.01). Means and standard errors are shown, *n* = 4, *n* = 5, and *n* = 4 for 1‐week, 3‐week, and adult control animals, *n* = 5, *n* = 6, and *n* = 5 for 1‐week, 3‐week, and adult sensitized animals, respectively.

Peripheral blood cell count showed a significant increase in eosinophils in neonatally sensitized adult animals compared with control adults (Fig. 3), whereas no statistically significant difference was observed in total leukocytes, monocytes, and neutrophils.

**Figure 4.**
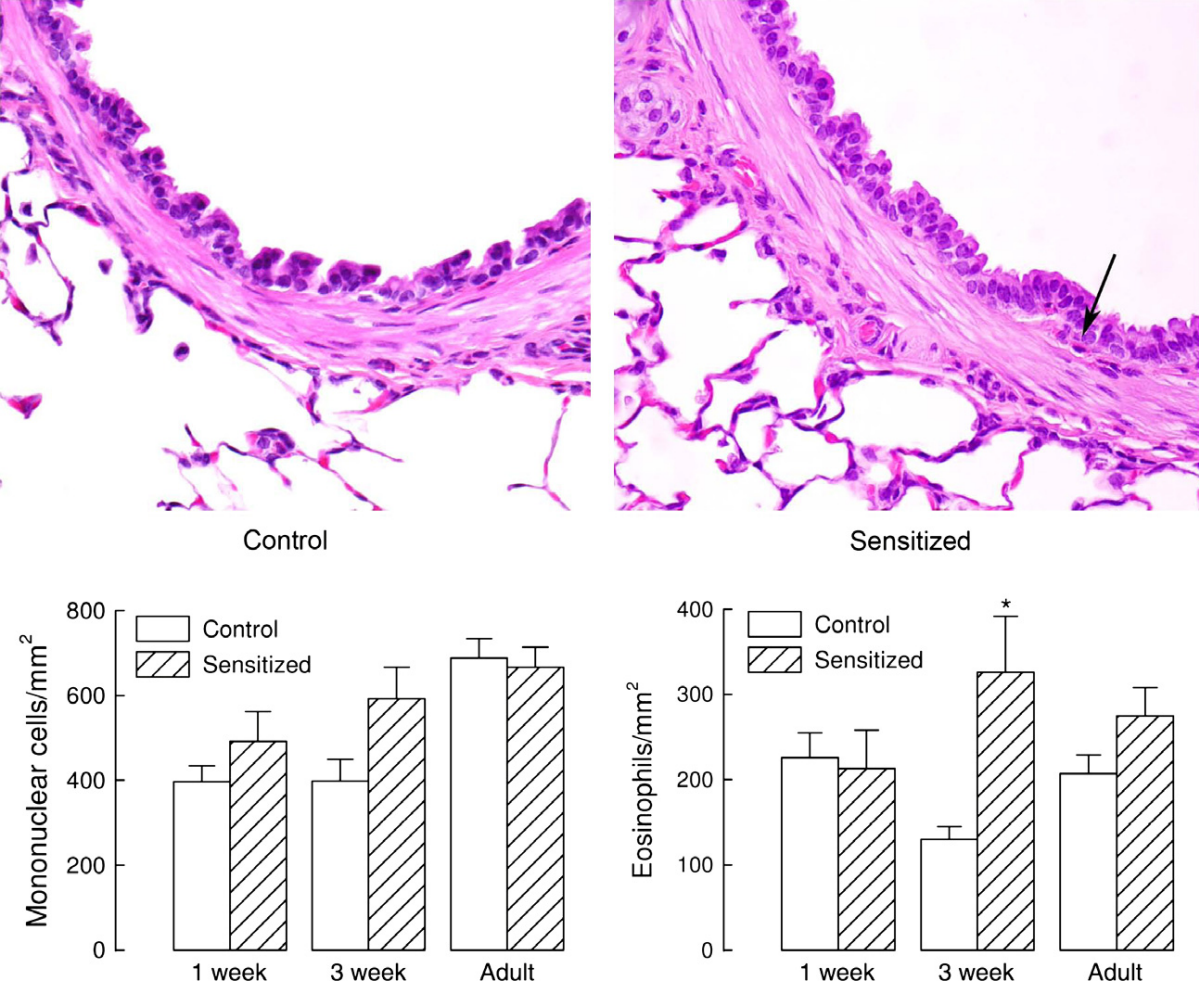
Inflammatory cells in the airway tissue. Histological analysis revealed absence of a prominent inflammatory process in the airway tissue from control guinea pigs and from animals sensitized at birth, except for an increased number of eosinophils in 3‐week animals neonatally sensitized compared with control animals. Representative slides of airway sections from adult animals stained with hematoxylin–eosin are shown in the top panels. The arrow shows an example of eosinophil in the airway wall. The number of eosinophils was significantly increased in the tissue of the 3‐week‐sensitized group compared to 3‐week controls (*P* < 0.05). Cell counts for mononuclear cells and eosinophils are shown in the bottom panels as means and standard errors, *n* = 5, *n* = 5, and *n* = 4 for 1‐week, 3‐week, and adult control animals, *n* = 5, *n* = 6, and *n* = 5 for 1‐week, 3‐week, and adult‐sensitized animals, respectively.

Tissue infiltration of inflammatory cells evaluated in the submucosa of large bronchi showed increased eosinophils only in 3‐week‐sensitized animals compared with controls of the same‐age group (Fig. 4), but not in comparison with the other sensitized and control groups.

**Figure 5.**
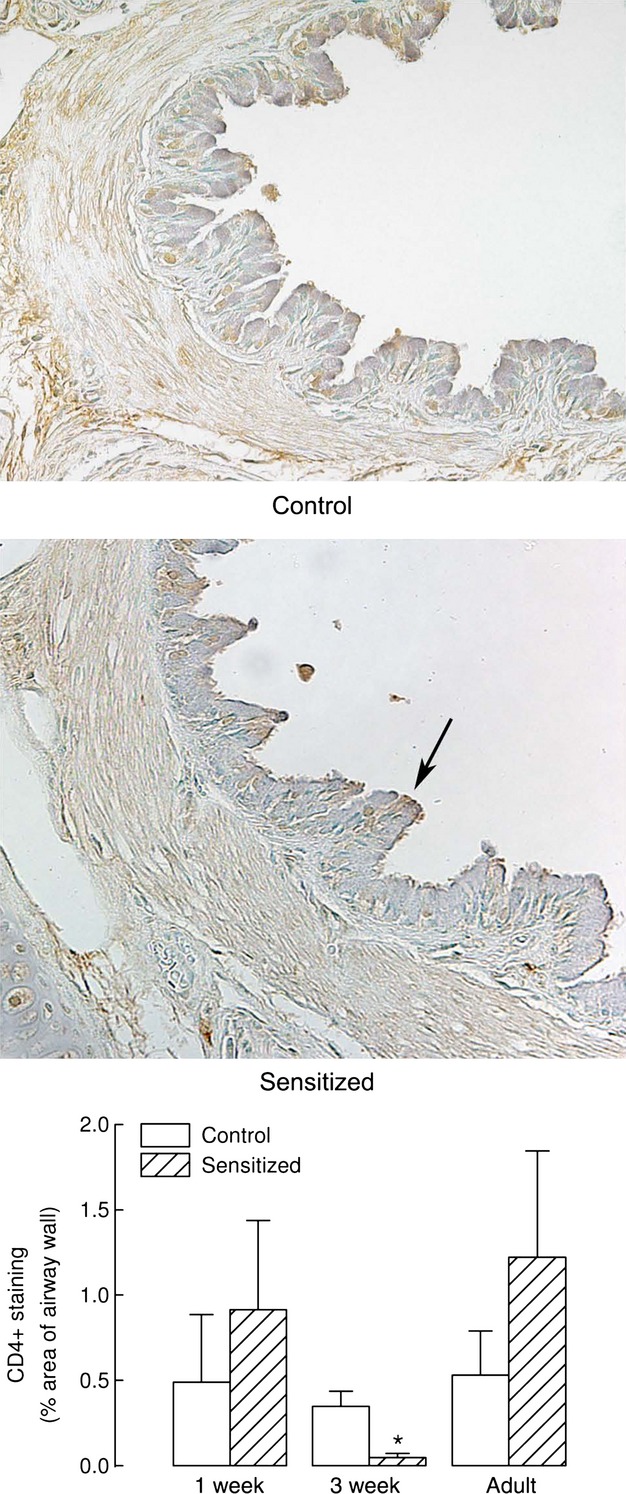
CD4+ staining. Immunohistochemistry staining for CD4+ cells was not increased in animals neonatally sensitized compared with control animals. Representative slides of airway sections from adult animals immunostained for CD4+ cells are shown in the top and middle panels. The arrow shows an area of the epithelium with intense positive staining for CD4. Computerized quantification of CD4+ staining, expressed as % area of the airway wall, in control guinea pigs and animals sensitized at birth is shown in the bottom panel as means and standard errors, *n* = 5, *n* = 5, and *n* = 5 for 1‐week, 3‐week, and adult control animals, *n* = 5, *n* = 6, and *n* = 5 for 1‐week, 3‐week, and adult sensitized animals, respectively. A significant reduction was observed in the tissue of the 3‐week‐sensitized group compared to 3‐week controls (*P* < 0.05).

Results of immunohistochemical tissue staining for CD4+ cells are shown in Figure 5. CD4+ staining was mainly localized within the epithelium. While a slight but not significant increase in CD4+ staining was found in large bronchi from 1‐week and adult‐sensitized animals, unexpectedly, we found a significant reduction of CD4+ staining in 3‐week‐sensitized animals.

### ASM active mechanical properties

#### Smooth muscle content

The content of smooth muscle in the total cross‐sectional area is a determinant of the active force generated in response to a given stimulus. Since sensitization may induce airway remodeling and modify ASM content, we measured the percent area of ASM in the airways of control and sensitized animals of the three age groups used in this study. We found no significant difference among our groups. The area of the airway wall occupied by ASM was, respectively, 32.1 ± 2.7, 30.5 ± 1.6, and 31.0 ± 1.1% in 1‐week, 3‐week, and adult control animals and 32.7 ± 1.2, 31.8 ± 2.1, and 29.5 ± 1.8 in sensitized animals. Therefore, an appropriate comparison of the generated force among different groups required only normalization to strip cross‐sectional area to account for different size of the strips.

#### Tone and stress generation

Table 1 shows the values of smooth muscle tone at rest and following electrical field stimulation, as well as the rate of stress generation. Resting tension was higher than the resting tension we reported in healthy guinea pigs at the same three ages (Chitano et al. 2000). The active tension, that is, the ability to generate force per mm^2^ of strip cross‐sectional area in response to electrical field stimulation, and the maximum rate of stress generation were comparable to the values we showed in healthy animals of the same three age groups (Chitano et al. 2000). No significant difference was found between sensitized tracheal strips from the three age groups. Smooth muscle tone in animals sensitized as adults was similar to what found in adult animals sensitized at birth. These data confirm that stress generation by ASM is not altered by allergen sensitization.

**Table 1.** Smooth muscle tone at rest and in response to electrical field stimulation

	1 week		3 weeks		Adult		
	Control	Sensitized	Control	Sensitized	Control	Sensitized	Sensitized as adults
*N*	10	9	11	14	14	11	6
RT (mN/mm^2^)	4.7 ± 1.1	8.9 ± 1.3	6.8 ± 0.7	9.1 ± 1.1	7.8 ± 2.8	10.9 ± 2.7	9.5 ± 2.4
AT (mN/mm^2^)	17.1 ± 1.9	17.5 ± 3.3	19.3 ± 3.8	17.0 ± 1.8	21.8 ± 2.3	17.0 ± 3.6	15.2 ± 2.3
RSG (AT/s)	0.40 ± 0.03	0.46 ± 0.04	0.45 ± 0.03	0.45 ± 0.02	0.51 ± 0.03	0.53 ± 0.02	0.55 ± 0.02

Resting tension (RT), active tension (AT), and rate of stress generation (RSG) in tracheal strips from different age guinea pigs sensitized to ovalbumin at birth or as adults and in controls. Data in control animals are from the experiments previously published in Chitano et al. 2000. Means and standard errors are shown, *N* is the number of animals.

#### Shortening velocity and power output

**Figure 6.**
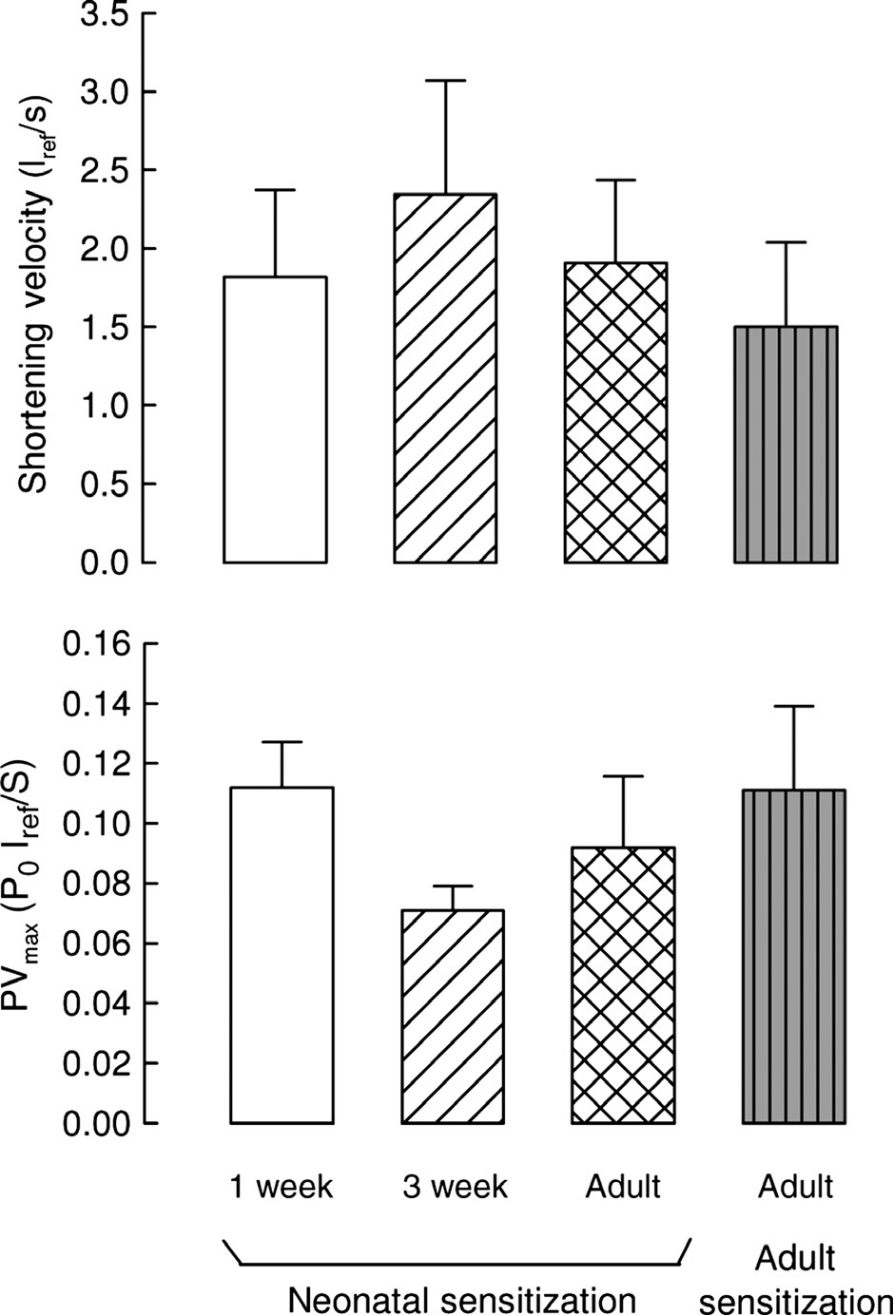
Shortening velocity and power. Neonatal ovalbumin sensitization prevented the considerable reduction of airway smooth muscle shortening velocity and power that occurs from 3 weeks to adulthood in healthy animals. Adult sensitization resulted in values of these mechanical parameters similar to neonatal sensitization. Maximum shortening velocity at zero load and maximum power (PV_max_) were measured in tracheal strips from different age guinea pigs sensitized to ovalbumin at birth or as adults. P_o_ is the maximum stress generated by EFS,* l*_*ref*_ is reference length. Means and standard errors are shown; *n* = 6, *n* = 9, and *n* = 9 for 1‐week, 3‐week, and adult animals neonatally sensitized, respectively; *n* = 6 for animals sensitized as adults.

Shortening velocity values in tracheal strips from neonatally sensitized guinea pigs are shown in the top panel of Figure 6. In our healthy control guinea pigs (Chitano et al. 2000), we showed a dramatic reduction of shortening velocity with maturation to adulthood: 1.79 ± 0.67, 2.45 ± 0.92, and 0.55 ± 0.09 *l*_*ref*_ /s in 1‐week, 3‐week, and adult tracheal strips, respectively. Following neonatal ovalbumin sensitization, the mean value of shortening velocity in adult tracheal strips was more than three‐fold the shortening velocity measured in control adults. Values at 1 and 3 weeks were nearly identical to those in control animals of the same age. As effect of neonatal ovalbumin sensitization, a significant difference in shortening velocity was no longer observed among the three age groups.

The maximal power produced by tracheal strips from neonatally sensitized animals is shown in the bottom panel of Figure 6. The power output in healthy control guinea pigs significantly decreased from 3‐week to adult, being, respectively, 0.084 ± 0.020, 0.149 ± 0.019, and 0.053 ± 0.007 P_o_·l_ref_/s in 1‐week, 3‐week, and adult (Chitano et al. 2000). Following early sensitization, power in adult strips was about two‐fold the power in adult controls, but was also surprisingly reduced by half it in the 3‐week group; as a result, the significant ontogenetic decrease of power present in tracheal strips from control guinea pigs was no longer observed in strips from sensitized animals.

In tracheal strips from animals sensitized during adulthood, both the shortening velocity (1.50 ± 0.54 *l*_*ref*_ /s) and the maximal power (0.111 ± 0.028 P_o_·l_ref_/s) were not significantly different from the values obtained in strips from adult animals sensitized at birth.

### Passive mechanical properties

#### Resistance to shortening

**Figure 7.**
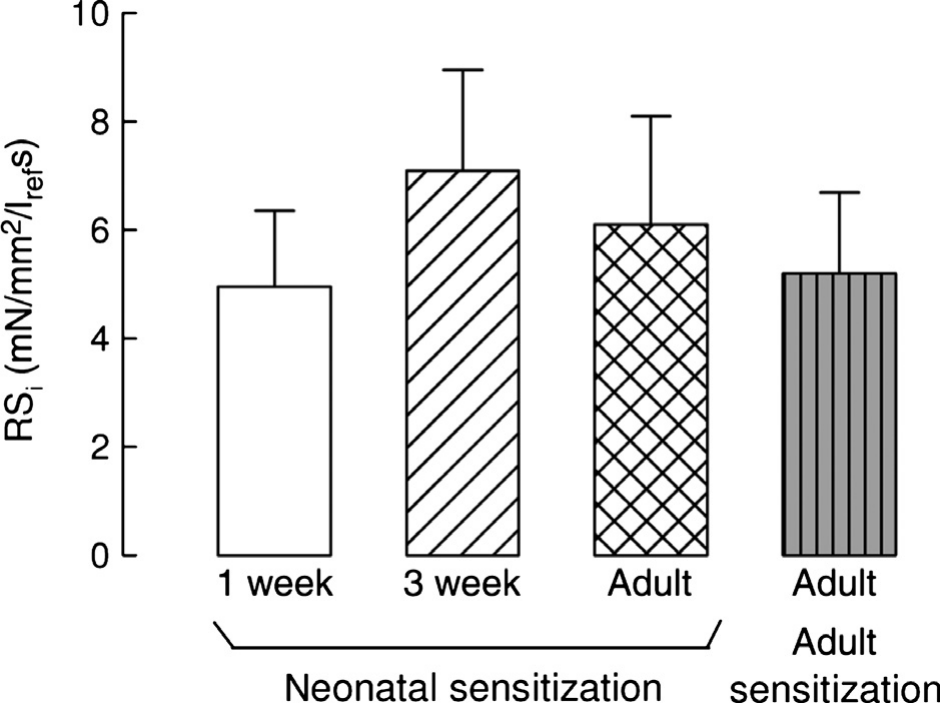
Resistance to shortening. Neonatal ovalbumin sensitization prevented the dramatic increase of airway smooth muscle internal resistance to shortening that occurs from 3 weeks to adulthood in healthy animals. Adult sensitization resulted in values of this mechanical parameter similar to neonatal sensitization. Internal resistance to shortening (RSi) was calculated from force–velocity experiments in tracheal strips from different age guinea pigs sensitized to ovalbumin at birth or as adults. Means and standard errors are shown, *n* = 6, *n* = 9, and *n* = 8 for 1‐week, 3‐week, and adult animals, respectively; *n* = 6 for animals sensitized as adults.

From the force–velocity curves used to measure shortening velocity, we calculated the internal resistance to shortening, RSi (Fig. 7). While the RSi in healthy control animals increased significantly in adult strips, being, respectively, 15.0 ± 5.2, 9.0 ± 3.0, and 32.1 ± 5.5 mN/mm^2^/l_ref_/s in 1‐week, 3‐week, and adult (Chitano et al. 2000), lower values of the RSi were observed after neonatal sensitization and no significant difference was found among the three age groups. These data show that neonatal sensitization prevented the increase of RSi that is associated with loss of the airway hyperresponsive phenotype in adulthood.

In strips from animals sensitized in adulthood, the RSi was 5.2 ± 1.5 mN/mm^2^/l_ref_/s, thus later sensitization reduced RSi to values similar to those in immature animals.

#### Vimentin content

**Figure 8.**
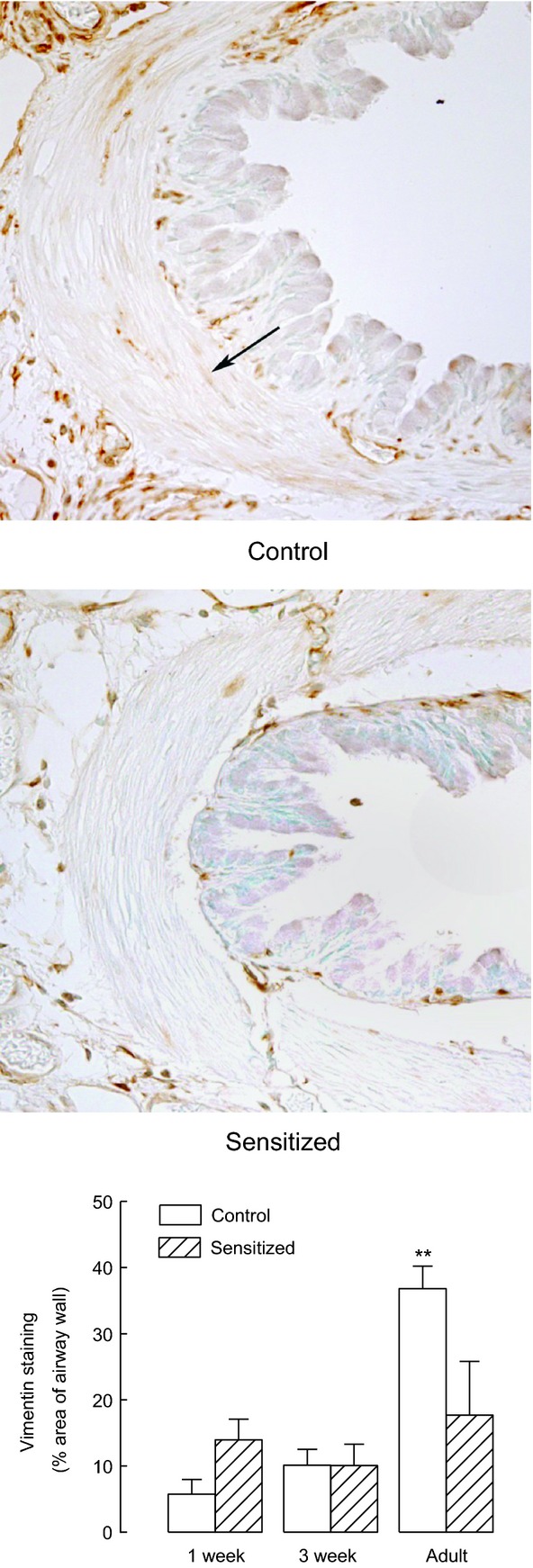
Vimentin immunostaining. Immunohistochemical staining for the cytoskeletal protein vimentin revealed a significant increase in adult control animals. This increase was prevented in animals neonatally sensitized to ovalbumin. Representative slides of airway sections from adult animals immunostained for the cytoskeletal protein vimentin are shown in the top and middle panels. The arrow shows an area of ASM with intense positive staining for vimentin. Sensitized adults had significantly less vimentin that adult controls (*P* < 0.01). Vimentin‐positive staining in the airways from control guinea pigs and from animals sensitized at birth, expressed as % area of the airway wall, is shown in the bottom panel as means and standard errors, *n* = 5, *n* = 5, and *n* = 4 for 1‐week, 3‐week, and adult control animals, *n* = 5, *n* = 6, and *n* = 5 for 1‐week, 3‐week, and adult sensitized animals, respectively.

Immunohistochemical analysis of the tissue content of vimentin, a fundamental cytoskeletal protein involved in cell and tissue mechanical stability, showed (Fig. 8) that a significant increase occurs in the airways of healthy animals at adulthood compared with younger animals (*P* < 0.01 by ANOVA) and that early sensitization prevented this ontogenetic increase (*P* < 0.01 comparing adult control and sensitized).

## Discussion

With this study we found that neonatal allergic sensitization, in the absence of subsequent allergen exposure, completely prevented the ontogenetic decline of ASM shortening velocity in adulthood as well as the simultaneous increase in resistance to shortening. We found no effect on the ability of ASM to produce force and on the content of ASM in the airway wall. We also found that ASM positive staining for vimentin increased with age in control but not in sensitized animals, similar to the changes we found for the internal resistance to shortening. These changes in vimentin and passive mechanical properties of the tracheal tissue provide evidence that allergic sensitization may affect airway responsiveness also by reducing the passive resistance that oppose the contractile response of the ASM. We observed a remarkable Schultz‐Dale response in animals sensitized at birth, whereas the response was less prominent when sensitization was done in adulthood. Since a mild inflammatory process, mainly characterized by presence of eosinophils in the blood and in the airway submucosa, was present in sensitized animals, an inflammatory response may be involved in the effects determined by neonatal sensitization.

The main feature of our model is the absence of any antigen exposure after the three injections employed to induce ovalbumin sensitization. This choice was based on the hypothesis that ASM hyperresponsiveness in sensitized individuals is not a consequence of allergen challenges but an intrinsic property of the sensitized airways. The Schultz‐Dale response in the 1‐week group (lower response to ovalbumin and several nonresponders) compared with the other two groups suggests that, following our sensitization protocol, the process of ASM sensitization is not completed at 1 week of age, but fully accomplished at 3 weeks. During the following 2 months, the extent of the ASM response to the specific antigen does not decline and the nonspecific hyperresponsive features are maintained at levels comparable to the 3‐week group. One important question prompted by our results is how long lasting both the specific response and the nonspecific hyperresponsiveness are. While it is well known that antigen avoidance alleviates allergic symptoms, it is not known whether it also reduces the magnitude of ASM‐specific and nonspecific contractile responses. Further investigation would be needed to evaluate the effect of time and aging on the persistence of ASM hyperresponsive phenotype following allergen sensitization in the absence of any additional allergen exposure. Because of the absence of any allergen challenge, we expected to find absence of inflammation in our sensitized animals. Nonetheless, our results showed an increased number of eosinophils in the airway wall of the 3‐week‐old animals neonatally sensitized and in the blood of adults, while no inflammation was produced by sensitization in the 1‐week‐old group. Increased number of eosinophils in the peripheral blood, alongside with increased number of eosinophils and mast cells in bronchoalveolar lavage fluid, was also reported in adult dogs sensitized at birth that were not exposed to the sensitizing agent for 2–3 months (Becker et al. 1989). That study did not measure inflammatory cells at earlier time points, while our data in 1‐ and 3‐week neonatally sensitized guinea pigs suggest that the increase in eosinophils does not occur immediately after allergen exposure. Based on these data, although we cannot reach a definitive conclusion, we suggest that an inflammatory process may be a delayed effect of neonatal sensitization that could contribute to the observed persistent airway and ASM hyperresponsiveness.

Young individuals are more susceptible to develop allergic reactions than adults as documented by the elevated incidence during childhood of diseases such as food allergy (Burks and Ballmer‐Weber 2006) and asthma (Martinez 2009). Moreover, especially during childhood, asthma is frequently associated with other allergic disorders such as eczema and allergic rhinitis (Zimmerman et al. 1988). Food allergy has been recognized as one of the best predictor of childhood asthma (Tariq et al. 2000). A strong correlation between diagnosed asthma and total serum IgE levels as well as between nonspecific airway hyperresponsiveness and IgE levels has been shown (Sears et al. 1991). Childhood eczema increases the likelihood of childhood asthma and its persistence to adulthood (Burgess et al. 2008). Exposure to house dust mite allergens in early life has been shown to be important for subsequent development of asthma (Sporik et al. 1990). In a canine model of atopically predisposed asthma development, exposure to allergen by inhalation from 1 to 20 weeks of age produced an asthmatic phenotype that was not observed when exposure was performed from 13 to 31 weeks of age (Royer et al. 2013). This body of evidence strongly supports the notion that sensitization to allergens in early life may be at the origin of the development of persistent airway hyperresponsiveness. Indeed, a study in dogs showed that in vivo nonallergic airway responsiveness, which gradually decreased between 4 and 15 months of age in control dogs, was significantly increased in 12‐ to 15‐month‐old animals neonatally sensitized to ragweed (Becker et al. 1989). In that study, animals were injected with the sensitizing agent within 24 h of birth, then weekly for the next 8 weeks and biweekly for other 8 weeks. They were then studied at 2‐month intervals and at each time point received one antigen inhalation challenge. Therefore, the measures of nonallergic airway responsiveness were performed 2 months after the last allergen exposure. Although it is not certain whether these periodical allergen inhalations may have affected the outcome of the study, the development of a chronic airway hyperresponsiveness was clearly demonstrated. Early life allergen exposure in a murine model has been shown to produce an increased ASM innervation and in vivo airway hyperreactivity that was not observed when exposure was performed in adult mice (Aven et al. 2014). In vivo nonspecific airway hyperresponsiveness has been also shown in adult animals that had early life viral infection (Dakhama et al. 2005; You et al. 2006; Schneider et al. 2012). Thus, the impact of early life events on maturation of airway function is not restricted to allergen sensitization, but it appears that several environmental insults occurring in the neonatal period may affect the normal ontogenesis of airway function and lead to its persistent impairment in adulthood.

Our results show that persistence of ASM hyperresponsive phenotype into adulthood may be an important contributor to the link between early allergic sensitization and persistent airway hyperresponsiveness later in life. In addition, ASM from neonatally sensitized individuals may acquire and/or maintain a more severe hyperresponsive phenotype than ASM from individuals sensitized as adult. Therefore, in one group of guinea pigs, we performed the sensitization protocol in adulthood and evaluated possible differences in comparison with neonatal sensitization. Our protocol induced sensitization in 100% of guinea pigs sensitized as adult and the average response to ovalbumin in ASM from animals sensitized as adults, although 2/3 lower, was not significantly different from the response in ASM from adult animals sensitized at birth. Also, active and passive mechanical properties of the ASM did not significantly differ between the two groups, suggesting that sensitization of adult airways produces a functional phenotype similar to the one observed in young healthy individuals as well as in adults sensitized at birth. We conclude that the effects of immune response on ASM in immature and mature airways are substantially similar, at least when the conditions of allergen exposure are prevalently oriented to a sensitization effect, as opposed to tolerance‐inducing conditions (Jun and Goodnow 2003). Whether adults require more stringent sensitizing conditions than young individuals to develop a hyperresponsive ASM phenotype is a matter that requires further investigation. Thus, adults may be less prone to become sensitized than young subjects, but acquire a comparable ASM hyperresponsiveness when they do become sensitized.

We found that allergic sensitization reduced the internal resistance to shortening, RSi, and the content of the cytoskeletal protein vimentin. Although the role of vimentin in airway hyperreactivity is not well defined, it is known that this cytoskeletal protein confers structural stability to the cell. Disruption of vimentin filaments results in loss of cell shape by destabilizing cytoskeletal interaction and cell substrate adhesion (Goldman et al. 1996). Thus, vimentin contributes to several mechanical properties of the cells, affecting contractility, motility, stiffness/compliance, and even cell growth (Eckes et al. 1998; Wang and Stamenovic 2000). Vimentin has also been shown to be involved in agonist‐induced force development in cultured airway smooth muscle cells, with reduced force associated with downregulation of filamentous vimentin (Wang et al. 2006). Those results could be explained by an involvement of vimentin in the mechanical coupling between contractile filaments and cell membrane that allows transmission of the generated force. Whether the activation‐induced function of vimentin is altered in allergen‐sensitized airway smooth muscle is currently not known. Our study confirm previous studies showing that allergic sensitization has little effects on force generation, suggesting that the changes in vimentin expression we observed with age in healthy guinea pigs may not be sufficient to modify the activation‐induced contribution of vimentin to force generation. Conversely, the increase in vimentin we found in adult healthy animals may modify sufficiently the overall cytoskeletal features to produce substantial changes of the mechanical properties of the cells with consequent increase of the RSi. Although additional studies are required to confirm that this is indeed a cause–effect phenomenon, based on this study, we suggest that neonatal allergen sensitization alone, that is, without any later exposure to the allergen, has the ability to produce long lasting, if not permanent, alteration of protein content in the airway tissue, which in turn may affect ASM/airway mechanics resulting in persistent airway hyperresponsiveness.

Our study provides additional evidence that, while force generation capability may not be a relevant factor, shortening velocity is a determinant component in ASM hyperresponsiveness (Jiang et al. 1994; Mitchell et al. 1994; Ma et al. 2002). Indeed, the unchanged ASM content and force‐generating ability we showed in this study confirms that ASM hyperresponsiveness following primary allergen exposure does not originate from the increased ASM mass typical of asthma (Carroll et al. 1993; Ebina et al. 1993; Kuwano et al. 1993) or content of contractile proteins. It has been shown that distinct cellular pathways are involved in the development of airway hyperresponsiveness following primary or secondary allergen exposure (Joetham et al. 2005); these distinct pathways conceivably produce dissimilar alteration in the ASM cells and in the airway tissue, originating hyperresponsiveness through distinct mechanisms. In a recent study, it has been shown that IgE increases the expression of MLCK at mRNA and protein levels in ASM cells, an effect that was inhibited by neutralizing antibodies against the high‐ affinity IgE receptor Fc*ε*RI (Balhara et al. 2014). This may likely be a mechanism by which a primary exposure to antigens, such as our neonatal ovalbumin sensitization, induces a hyperresponsive phenotype in ASM. Several other mechanisms could also be at the origin of our observations and remain to be investigated to understand, for example, whether neonatal allergen sensitization induces contractile alterations that involve GPR signaling, calcium mobilization, myosin light chain phosphatase activity by effect of calcium sensitization and other relaxation factors, or structural modifications involving extracellular matrix or cytoskeletal proteins. We suggest that our finding of lower vimentin content associated with increased ASM shortening velocity and power output in allergen‐sensitized adult animals compared to adult controls is a potential mechanism regulating ASM hyperresponsiveness.

In conclusion, we have shown that allergic sensitization at birth, without any subsequent allergen exposures, is sufficient to induce persistence to adulthood of the immature ASM hyperresponsive phenotype. This includes elevated ASM shortening velocity and low values of RSi. Allergic sensitization in adults induces a similar phenotype. We suggest that the specific ASM and/or airway physiological/immunological features in early life may facilitate the induction by early allergen exposure of a severe permanent hyperresponsive ASM/airway phenotype later in life. The persistence of this hyperresponsive phenotype does not require further direct airway exposure to allergen.

## Acknowledgments

The authors thank Dr. Carrie M Cox for her help in developing the early sensitization protocol and Dr. Janet A Jenkin for her help in setting a computerized approach to the analysis of experimental records. The results of one set of experiments contained in this work were presented in preliminary form at the 1999 meeting of the American Thoracic Society. Preliminary results of this work were also presented at the International Symposium on Recent Research Advances in Asthma Pathogenesis, held November 2006 in St. John's, Antigua.

## Conflict of Interest

The authors have no conflict of interest.
